# The Relevance of the Interfacial Water Reactivity for Electrochemical CO Reduction on Copper Single Crystals

**DOI:** 10.1021/acscatal.3c02700

**Published:** 2024-01-08

**Authors:** Daniel Winkler, Matthias Leitner, Andrea Auer, Julia Kunze-Liebhäuser

**Affiliations:** † Department of Physical Chemistry, University of Innsbruck, Innrain 52c, 6020 Innsbruck, Austria

**Keywords:** Cu(100), Cu(111), CO electroreduction, STM, water reactivity, DEMS, HER. (Min.5-Max. 8)

## Abstract

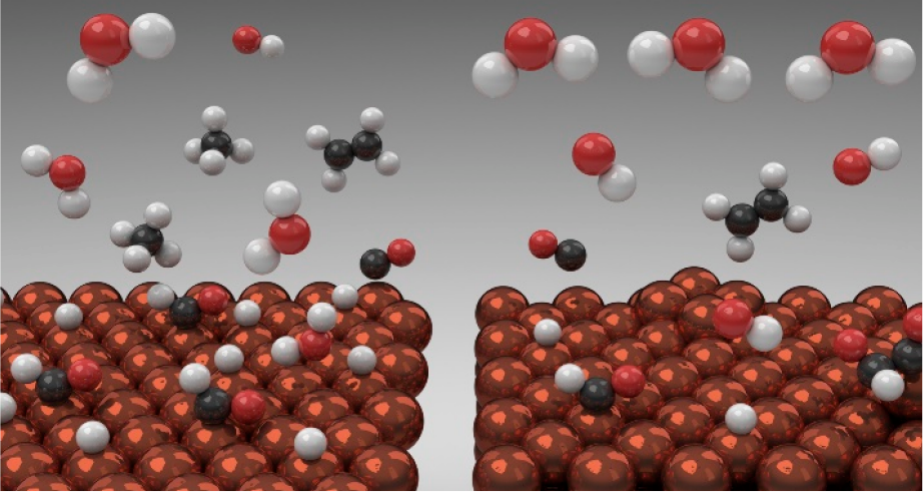

The electrochemical reduction of CO_2_ is an important electrolysis reaction that enables the conversion of a waste gas to fuels or value-added chemicals. To make this reaction viable, a profound understanding of central intermediate steps, such as the CO electroreduction, is required. On Cu, the CO reduction reaction (CORR) is intimately linked to the hydrogen evolution reaction (HER) that proceeds via the reduction of water in alkaline or neutral electrolytes. Here, we demonstrate that the interaction of water or more specifically the water reduction kinetics on differently smooth Cu(100) and Cu(111) surfaces during the CORR in alkaline media significantly governs the CORR. On Cu(111), faster HER kinetics and the highest CORR activity are observed, even though HER and CORR onsets are more negative. While on Cu(100) small Cu ad-island clusters form in the cathodic potential range only when CO is present, structural changes appear on a larger length scale on Cu(111) both under CORR conditions and when no CO is present. These differences in the reconstruction characteristics may be attributed to the dominance of either the CORR and its intermediates or the HER on the different Cu surfaces. Therefore, the interfacial water reactivity is considered an essential activity descriptor for the CORR on Cu in alkaline media.

## Introduction

For the sustainable production of chemical feedstocks and fuels, the electrochemical CO_2_ reduction is considered an auspicious approach.^1^ This type of CO_2_ utilization enables its depletion from the earth’s atmosphere and renewable electricity storage. Different (transition) metal electrodes have been tested as electrocatalysts for the CO_2_ reduction reaction (CO_2_RR) and can be categorized in four different groups depending on the main CO_2_RR products.^2^ Cu is the only known pure metal that catalyzes the formation of hydrocarbons, i.e., methane (CH_4_) and ethylene (C_2_H_4_) with reasonable Faraday efficiencies as well as smaller amounts of alcohols.^1−4^ The design of energy efficient and product selective catalysts for the CO_2_RR remains a challenging task due to the complexity of the reaction, which depends on, e.g., the (surface) pH, electrolyte composition, and/or the chemical nature and atomic structure of the catalyst surface.^1,5,6^ All key factors for the course of the electrochemical CO_2_RR on Cu electrodes in aqueous electrolytes, such as electrolyte composition and pH, but also electrode morphology and surface structure, that impact the reaction pathway, mechanism, and rate have been comprehensively reviewed by Nitopi et al.^1^ In this review, most of the current knowledge of the various parameters influencing copper’s activity and product distribution is outlined in great detail. The structure–activity relations of single crystalline Cu for the CO_2_RR have been recently reviewed in ref (7). It is known that on Cu electrocatalysts both product distribution and CO_2_RR activity depend on the different surface sites and crystallographic orientations. The currently common perception is that, on single-crystalline Cu(111), CH_4_ formation has the highest rate, and only small amounts of C_2_H_4_ are produced, while on Cu(100), C_2_H_4_ is primarily formed.^3,4,8^ Cu(100) with an increased number of steps and defects has been reported to produce even more C_2_H_4_ than well oriented mostly planar (100) surfaces.^1^

The introduction of surface defects also strongly influences the catalytic activity. This is heavily discussed in the literature due to the undeniable impact of the surface roughness and defect site concentration, induced by the Cu electrode pretreatment, on the CO_2_RR activity.^9^ This pretreatment comprises conventional single-crystal preparation, i.e., mechanical and electrochemical polishing, annealing, and transfer. It was recently shown that clean and atomically ordered Cu(111) and Cu(100) are dominated by the HER under CO_2_RR conditions, while surface roughness and defects, induced through electropolishing and plasma treatments, favor the formation of hydrocarbons.^5^

The HER plays a crucial role in the CO_(2)_RR as a pervasive side reaction apparently diminishing the overall formation efficiencies of value-added CO_2_ reduction products and generally influencing the CO_2_RR mechanism through its activity at the interface. It is generally accepted that the reaction pathways toward CH_4_ and C_2_H_4_ formation differ after *CHO intermediate formation (where “*” denotes adsorbed species here and in the following), where the presence of adsorbed hydrogen (H*) and *CO species plays a major role, because the coupling of CHO with hydrogen leads to CH_4_, coupling with CO to C_2_H_4_ formation.^10^ Thus, the adsorption of hydrogen plays a key role in the CO_2_RR mechanism and is likely responsible for product selectivity. As shown in theoretical considerations, the special role of Cu lies in showing ideal CO and H adsorption energies.^11^ At present, many different theoretical models assume the importance of either adsorbed H* or H_2_O* for the nature of the products and the selectivity.^11−14^ Hydrocarbon formation from CO involves the transfer of protons or adsorbed hydrogen from the surface and is, thereby, strongly dependent on the water dissociation.

The reactivity of the interfacial water is therefore central for this step beyond CO, especially under alkaline or intermediate pH conditions, as it is for the alkaline HER.^15,16^ It has been shown, that in alkaline electrolytes, the adsorption of hydrogen and the HER and CO_2_RR kinetics are slower than in acidic media, because they take place far away from the potential of zero free charge (pzfc), where interfacial water is less reactive, as demonstrated for Pt^15−17^ and for Cu.^18,19^ Through modification of interface water, the overall efficiency of the CO_2_RR could be increased,^20^ which leads to the assumption that the HER onset and kinetics have a major influence on the reaction pathway and kinetics of the CORR.

Cu(111) and Cu(100) fundamentally reconstruct at moderate HER current densities in acidic media.^21−24^ This suggests that their structure is also altered during the CO_2_RR, which was not clearly demonstrated until very recently,^25−27^ because in most previous studies no CO/CO_2_ was inside the electrolyte.^26,28−30^ During the CO_2_RR in CO_2_-saturated 0.1 M KHCO_3_, surface roughening has been imaged with electrochemical atomic force microscopy (EC-AFM).^27^ Recent EC-STM studies in combination with Raman spectroscopy and X-ray diffraction of Cu(100) in 0.1 M KHCO_3_ show that the formation of CO from CO_2_ via adsorbed carboxylate intermediates induces the formation of Cu nanoclusters in the potential range of CO_2_ reduction.^25^ In this system, (bi)carbonate is present as an adsorbate layer at the Cu(100) surface and strongly hinders the adatom mobility at the surface.^25^

In this work, we study the electrochemical CO reduction on Cu(100) and Cu(111) in 0.1 M NaOH (pH 13). We combine online product determination via differential electrochemical mass spectrometry (DEMS), with a focus on the main gaseous products H_2_, CH_4_, and C_2_H_4_; analysis of adsorbed intermediates with electrochemical infrared reflection absorption spectroscopy (EC-IRRAS); and imaging of structural changes with electrochemical scanning tunneling microscopy (EC-STM). All methods are employed under potential control *in situ* during the CORR. DEMS gives macroscopic information on the onset potentials of the three products and on the relative activities toward HER and CORR and provides insight in the hydrogen evolution and adsorption kinetics. Both HER and CORR are governed by the reactivity of interfacial water that determines the nature of adsorbates and the interface chemistry as probed with EC-IRRAS. To get a microscopic picture of the surface and its structural changes during the CORR, we monitor the *in situ* structure evolution at potentials where hydrocarbons are formed with EC-STM. Both surfaces reconstruct on the atomic scale, where on Cu(100) small Cu ad-islands form immediately when the potential is stepped to values where C_2_H_4_ is produced, while Cu(111) shows larger morphological changes under the same conditions. While similar changes appear on Cu(111) in the CO-free electrolyte, Cu(100) shows no formation of ad-islands. This highlights the strong interaction of interfacial water with the Cu(111) surface, whereas on Cu(100) the interaction of the CORR intermediates dominates.

## Results and Discussion

**Figure 1:**
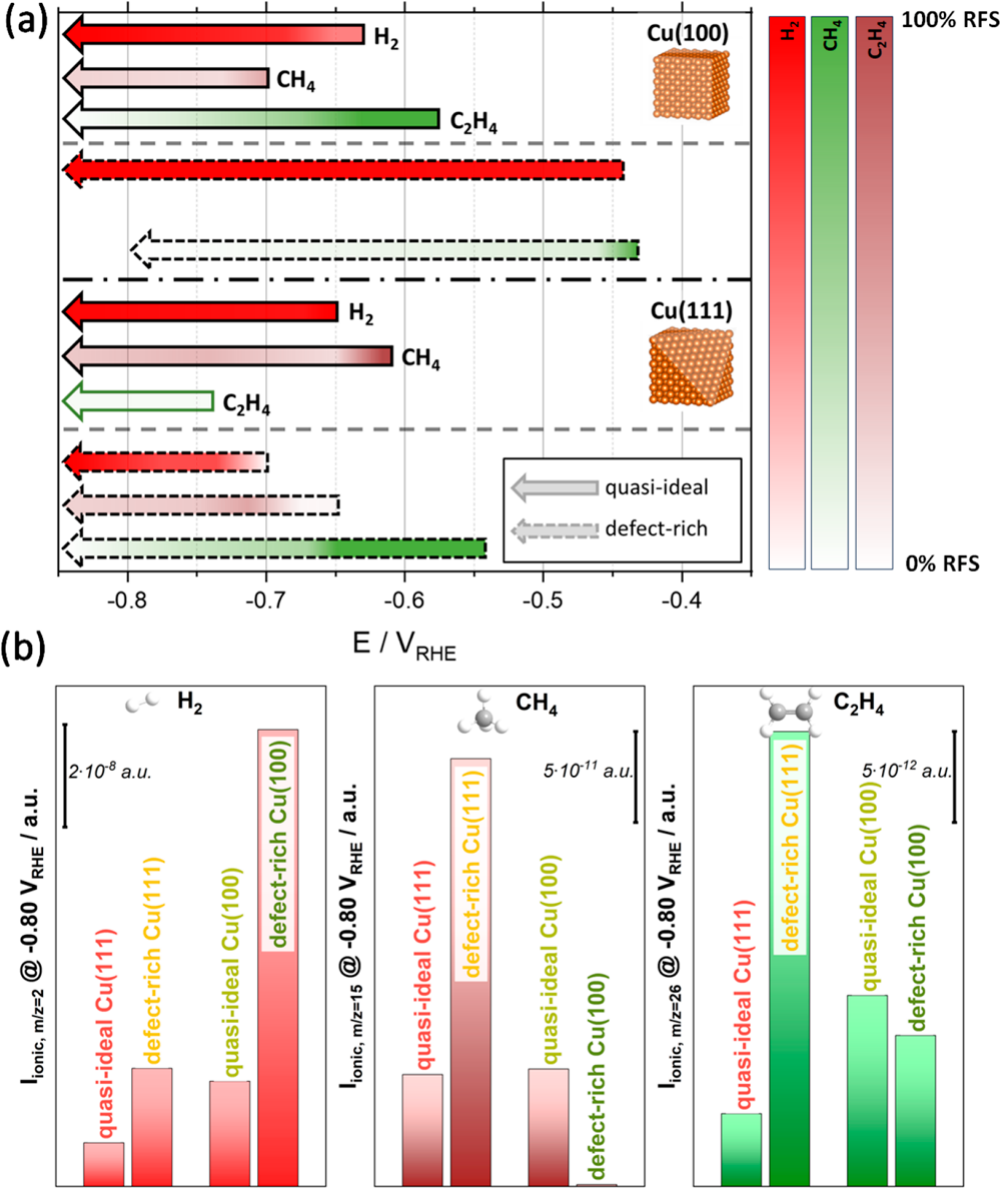
(a) Potential window (arrows) and relative Faraday selectivities (color intensity) for H_2_, CH_4_, and C_2_H_4_ formation for quasi-ideal (full lines) and defect-rich (broken lines) Cu(100) and Cu(111). (b) Relative activities from the ionic currents of the *m*/*z* signals 2 (H_2_), 15 (CH_4_), and 26 (C_2_H_4_) at −0.80 V_RHE_.

The electrochemical activity and distribution of the dominant gaseous products^6^ formed during CO reduction on Cu(100) and Cu(111) in 0.1 M NaOH is first assessed through voltammetry in combination with online DEMS. This technique allows the clear identification of the formation onset potentials of all products (see Supporting Table S1) and a semiquantitative determination of the product formation activities and selectivities, due to the correlative nature of the product amounts and the respective ionic currents.^31^ It is noteworthy that due to different collection efficiencies, ionization probabilities, and ion-to-electron conversion efficiencies, all given ionic currents are relative not absolute quantities; their utilization is common practice in the case of DEMS data evaluation for the CO_(2)_RR,^31−33^ where product calibration is most challenging. The ionic currents reported in the present paper compare well with the values that are usually measured with single crystals in DEMS (flow) cells.^33,34^ Since surface defects induced by the pretreatment of the crystal, as described in ref (5), play a crucial role for the CORR activity, annealed (“quasi-ideal”) and electropolished (“defect-rich”) Cu surfaces were examined in terms of their catalytic activities. These surface types are prepared according to standard procedures generally used for electrochemical investigation.^5,6,33^Figure 1a shows the potential windows where the three main gaseous products, H_2_, CH_4_, and C_2_H_4_, are detected (arrows in Figure 1a), as determined from the online DEMS results shown in Supporting Figures S1 and S2. Additionally, the relative Faraday selectivities (RFS) are provided by the color intensities in Figure 1a and in Supporting Figure S3. Figure 1b shows the ionic currents of the *m*/*z* signals for H_2_, CH_4_, and C_2_H_4_, at −0.80 V_RHE_ as a measure of their relative formation activities at that potential. On quasi-ideal Cu(100), the HER starts at about −0.63 V_RHE_, with CH_4_ formation at slightly more negative potentials (−0.70 V_RHE_), while C_2_H_4_ formation has an earlier onset at −0.56 V_RHE_ (see Figure 1a and Supporting Figures S1 and S4). The C_2_H_4_ formation stagnates at around −0.75 V_RHE_, while the ionic currents for CH_4_ and H_2_ keep increasing exponentially (see Supporting Figure S1). On defect-rich Cu(100), no CH_4_ formation is observed in the measured potential window, while the HER and C_2_H_4_ production are significantly enhanced and start at lower onset potentials of −0.44 and −0.43 V_RHE_, respectively (see Figure 1, Supporting Figures S1 and S4). This corresponds to an overpotential decrease by ∼0.2 V for the HER and by ∼0.1 V for ethylene formation upon increasing the defect sites. This early C_2_H_4_ formation onset perfectly agrees with that reported in ref (33) and is commonly justified by the presence of defects at the surface^27^ that lead to an enhancement of the C_2_H_4_ formation at low overpotentials.

Quasi-ideal Cu(111) shows rather weak relative HER activity (see Figure 1b and Supporting Figure S2), and CH_4_ is produced in comparable amounts as on quasi-ideal Cu(100) (see Supporting Figure S2 and S1); the onsets are at −0.65 and −0.61 V_RHE_ in accordance with the literature.^4,35^ On defect-rich Cu(111), the relative HER activity increases remarkably, and the amount of formed CH_4_ more than doubles (see Figure 1b and Supporting Figure S2); the respective onset potentials are at −0.70 and −0.65 V_RHE_. The C_2_H_4_ formation activity is the highest of all investigated samples with an onset at −0.54 V_RHE_ (see Figure 1 and Supporting Figures S2 and S4). Since the ionic currents directly correlate with the activity of a given catalyst, defect-rich Cu(111) is considered the most active among all investigated surfaces for the CO electroreduction toward hydrocarbons.

**Figure 2:**
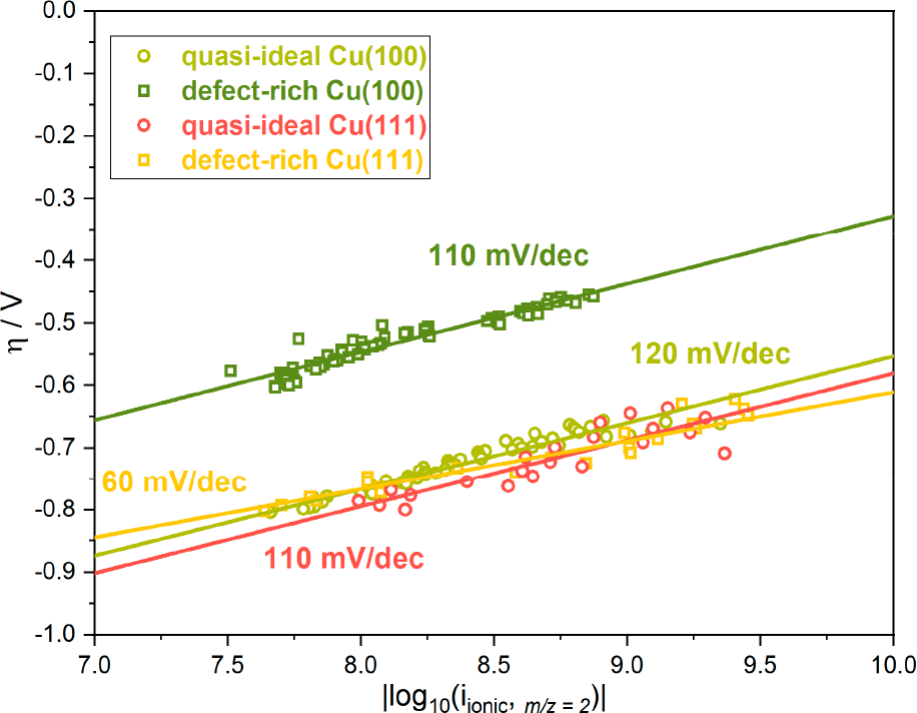
Tafel plots for the HER derived from the ionic currents of hydrogen (*m*/*z* = 2) measured with DEMS for quasi-ideal and defect-rich Cu(100) and Cu(111) in 0.1 M NaOH. Scan rate 2 mV/s.

We attribute the observed highest overall activity toward C_2_H_4_ formation on defect-rich Cu(111) to the later H_2_ evolution onset, whereas on defect-rich Cu(100) the strong H_2_ formation intensity reduces the overall activity toward C_2_H_4_ formation. The earliest HER onset is observed for defect-rich Cu(100), and the latest is observed for defect-rich Cu(111). Most interestingly, in CO-free 0.1 M NaOH, the HER onsets (see Supporting Table S2 and Figure S5) occur at the same potentials, where C_2_H_4_ formation begins in a CO-saturated electrolyte (−0.42 V_RHE_ on Cu(100) and −0.54 V_RHE_ on Cu(111)). As theoretically predicted,^11,35^ this leads to the assumption that the hydrogen adsorption is a central factor for the formation of hydrocarbons. H* formed on the surface initiates the HER in a CO-free electrolyte, whereas when CO is present, the HER is suppressed because CO is preferentially hydrogenated by H* to *CHO at these low overpotentials.^13,14^ Key parameters in this context are the hydrogen adsorption probability and coverage, the latter of which can be deduced from the HER kinetics^36^ that are accessible via the Tafel slope analysis of the ionic hydrogen currents at *m*/*z* = 2 from the DEMS data in this study.^37,38^ The Tafel plots of the four different Cu surfaces in CO-saturated NaOH are shown in Figure 2. The corresponding reference measurements in the CO-free electrolyte, where, intriguingly, the HER kinetics are generally slightly slower (120–130 mV/dec in all four cases), are shown in the Supporting Information (Supporting Figure S6). For quasi-ideal Cu(111) and both Cu(100) surfaces, the slopes are ∼110–120 mV/dec, suggesting that the formation of adsorbed H* (Volmer-Step) is the rate-determining step (RDS).^40,41^ For defect-rich Cu(111), the fastest HER kinetics, with a Tafel slope of 60 mV/dec, are measured, which indicates a Volmer–Heyrovsky limitation; i.e,. on most active sites not the hydrogen adsorption but the reaction of adsorbed hydrogen with water from the electrolyte (Heyrovsky step) is the RDS, whereas on some sites still the hydrogen adsorption (Volmer step) is rate limiting.^36,37,39^ It can thus be concluded that, among the four selected surfaces, defect-rich Cu(111) provides the lowest barrier for the hydrogen adsorption and thus the highest hydrogen coverage.^36^ Since the general hydrocarbon formation activity is also maximal on defect-rich Cu(111), we conclude that adsorbed H* is in fact essential for the formation of the *CHO intermediate,^10^ which in turn is needed for hydrocarbon formation.^10−12^ It is known that the reaction pathways to either C_2_H_4_ or CH_4_ also depend on the chemical surroundings of the *CHO intermediate, specifically on the amount of adsorbed H*.^10^ Accordingly, a higher C_2_H_4_ selectivity is reached with only small amounts of available H*, which makes *CHO react with a CO molecule to form C_2_H_4_ rather than with *H to produce CH_4_. Although high relative C_2_H_4_ activities are measured for defect-rich Cu(111), the comparably high amounts of H* can also enhance the reaction toward CH_4_ formation at higher overpotentials.

**Figure 3:**
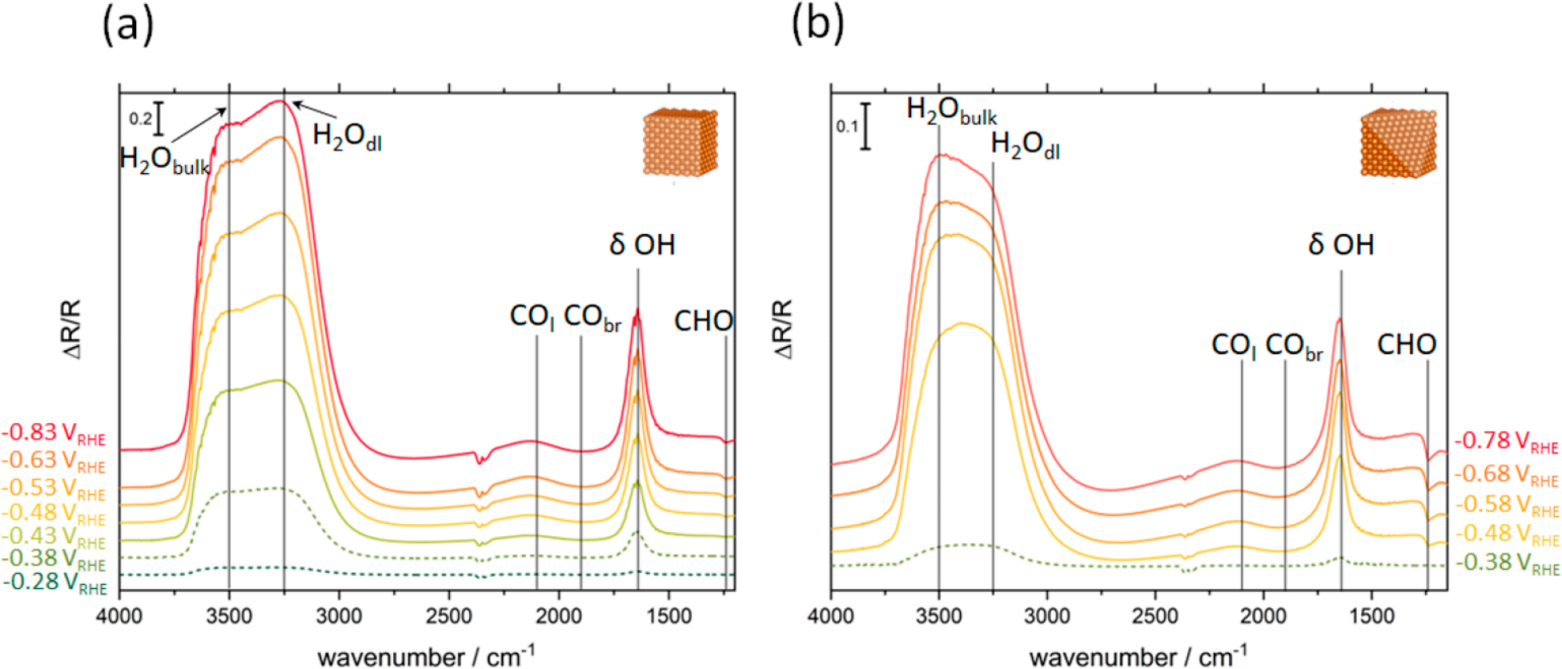
EC-IRRA spectra in CO-containing 0.1 M NaOH of (a) Cu(100) and (b) Cu(111). All spectra are referenced to *E*_ref_ = −0.18 V_RHE_. Spectra in dashed lines are obtained at more positive potentials than the CORR onset.

Since hydrogen adsorption proceeds via the dissociation of water (H_2_O) in alkaline media,^15,16,36^ it is evident that the interfacial water and its reactivity play a central role in the CORR. To probe the interactions of CO and H_2_O with the surface, *in situ* EC-IRRAS was conducted on Cu(111) and Cu(100). Since the focus of EC-IRRAS lies on mechanistic understanding, no distinction between defect-rich and quasi-ideal Cu(*hkl*) will be made in the EC-IRRAS section. The EC-IRRA spectra are shown in Figure 3, where the formation (downward bands) and the consumption (upward bands) of interface species as a function of the applied potential are depicted. Small bands of atmospheric CO_2_ appear at 2350 cm^–1^, due to the presence of atmospheric air in parts of the spectrometer’s optics. In both spectra, no adsorbed CO, either linearly or bridge bound,^40^ is visible in the investigated potential window. This contradicts most CO_2_RR studies conducted in neutral carbonate buffer electrolytes, where CO adsorbates are readily visible at the Cu surface. A recent study of the CO_2_RR on Cu(100)^25^ provides a very sound explanation for this by revealing that the CO adsorbate is formed via reduction of carboxylate intermediates. Instead of adsorbed CO, we detect a band at 1240 cm^–1^ after the CORR onsets on both Cu(100) (−0.4 V_RHE_) and Cu(111) (−0.5 V_RHE_; see Figure 3 (solid lines), Figure 1, Supporting Figures S1 and S2, and Supporting Table S1), which we attribute to hydrogenated CO (*CHO) according to the literature.^41^ This can be explained by the very fast hydrogenation of CO to *CHO.

Adsorbed H* seems to be essential for this *CHO formation, since the intensity of the CHO signal is 2 times higher in the IR spectra of Cu(111), which provides a higher H* adsorbate coverage than Cu(100), according to the Tafel slope (see Figure 2). The reactivity of the interfacial water, which influences the kinetics of the H* adsorption and consumption, is evident from the water band signals at ∼2800–3750 cm^–1^, even though the high absorbance of water makes the interpretation of these bands challenging. Following the recent interpretation by Cuesta et al.,^20,42,43^ the different water specimens are distinguishable by their wavenumbers as water in the electrical double layer (H_2_O_dl_) at ∼3250 cm^–1^ and bulk water (H_2_O_bulk_) at higher wavenumbers. H_2_O_bulk_ can either be bound to one to three hydrogen atoms (∼3500 cm^–1^) or have a very low degree of H-bonding (∼3600 cm^–1^).^20,42−44^ At potentials after the CO reduction onset (solid lines in Figure 3), a preferential consumption of either H_2_O_dl_ or H_2_O_bulk_ is visible through the comparison of the intensity ratios between the IR bands at ∼3250 cm^–1^ (H_2_O_dl_) and at 3500–3600 cm^–1^ (H_2_O_bulk_). For Cu(100), the depletion of H_2_O_dl_ is more pronounced than that of H_2_O_bulk_, a trend starting at −0.43 V_RHE_, which is the onset potential of the CORR and HER determined by DEMS. This is also in accordance with the Tafel slope analysis, which suggests that the Volmer step is rate determining, i.e., the recovery of H_2_O_dl_ is comparatively slow, leading to lower hydrogen coverages.

Despite the early HER onset, in the case of defect-rich Cu(100), the low reactivity of interfacial water causes H_2_O_dl_ to be preferentially consumed and slowly recovered, leading to slow HER kinetics. On Cu(111), a preferential depletion of H_2_O_bulk_ is visible and the consumption of H_2_O_dl_ appears less dominant at potentials < −0.58 V_RHE_, i.e., close to the CORR onset. This confirms the easier recovery of H_2_O_dl_ on Cu(111) compared to Cu(100), which is likely due to a higher reactivity of the interfacial water,^16^ which causes an increased H* coverage and leads to higher CO conversion through efficient hydrogenation of CO to *CHO.

**Figure 4:**
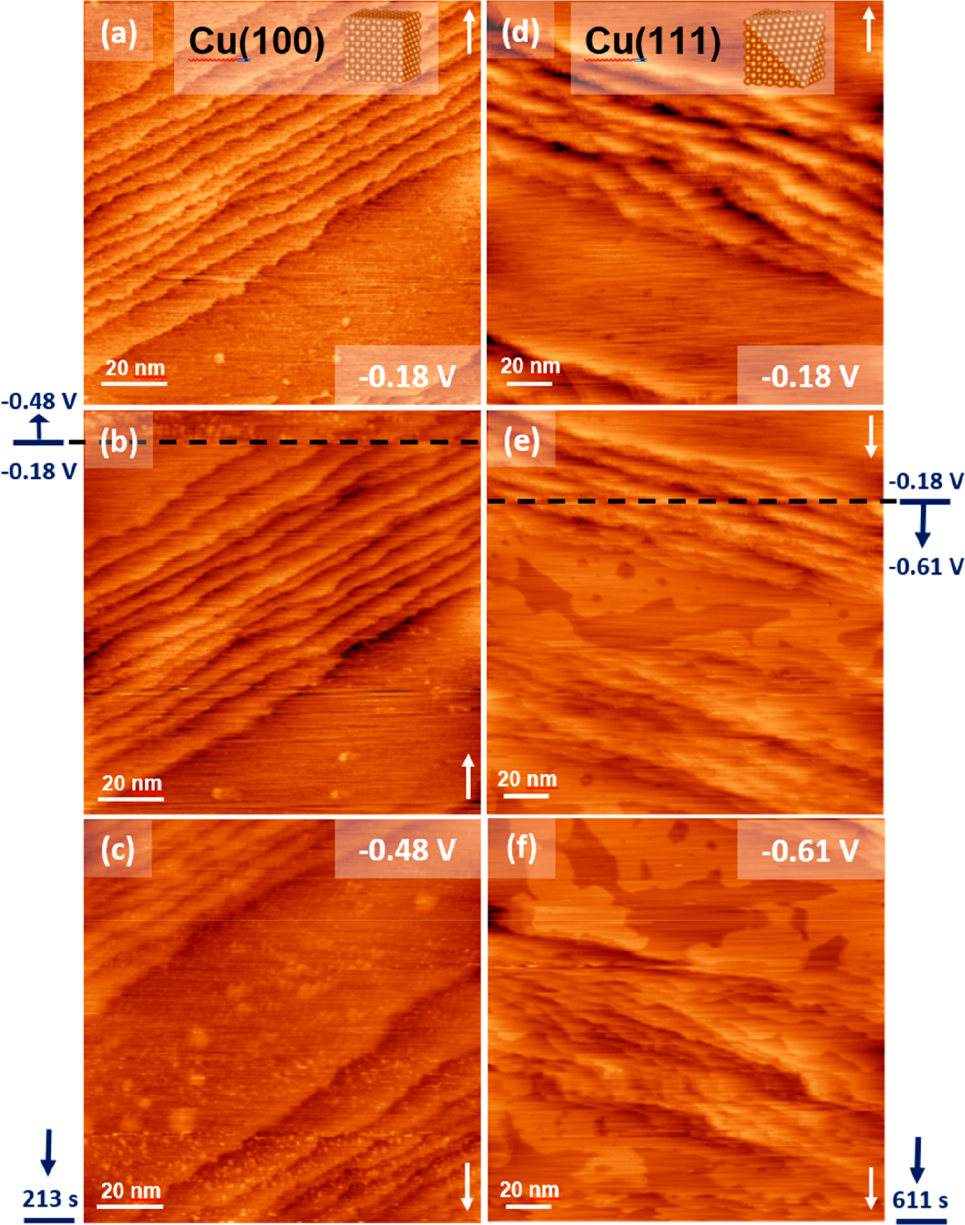
EC-STM images of Cu(100) (a–c) and Cu(111) (d–f) during potential steps (black dashed lines) in CO-saturated 0.1 M NaOH from the reference potential −0.18 V_RHE_ to the range of the CORR at −0.48 and −0.61 V_RHE_, respectively. Slow scan directions are indicated with white arrows in the top and bottom right corners of the images. Image sizes: 120 × 120 nm^2^, *I*_tun_ = 1.8 nA, *E*_tip_= 0.35 V_RHE_; Cu(111) in d–f, image sizes: 180 × 180 nm^2^, *I*_tun_ = 2.8 nA, *E*_tip_= 0.28 V_RHE_. Elapsed times after the potential steps are given in the left and right lower corners.

Profound reconstruction of Cu has been reported under HER conditions,^21−23^ and surface roughening, most likely due to restructuring has also been reported at CO_2_RR potentials.^25,27^ Since the variation of defect concentration and types certainly influences the reaction pathway and mechanism of the CO_(2)_RR, it is of the utmost importance to monitor surface morphology and structure evolution under the reaction conditions. To accomplish this, *in situ* EC-STM was conducted at the CORR onsets, i.e., at −0.48 V_RHE_ on Cu(100) and −0.61 V_RHE_ on Cu(111). The respective EC-STM images are shown in Figure 4. Cu(100), as the surface with the earliest CO reduction onset, shows an immediate formation of small ad-islands with the most common diameters of 0.52, 1.35, and 2.19 nm upon a potential step in the range where C_2_H_4_ formation starts (−0.48 V_RHE_; see Figure 4a–c and Supporting Figure S7). The small islands remain at the surface as long as the potential is held at −0.48 V_RHE_ and also tend to form larger agglomerates under CORR conditions (see Figure 4c and Supporting Figure S8a,b). When the potential is stepped back to −0.18 V_RHE_, the ad-structures are still stable over a prolonged period of time. Due to the low Faraday currents at that potential, all the features are better identifiable: ad-islands, ad-island agglomerates in the form of patches and chains, as well as small holes are visible at the surface (see Supporting Figure S8b,c). The ad-islands blur out and disappear slowly, and terraces smoothen after an extended period of time (∼3700 s) at the reference potential of −0.18 V_RHE_ (see Supporting Figure S8d).

On Cu(111), pronounced structural and morphological rearrangements are also observed upon the potential step into the range of CO reduction, but the visible changes occur on a larger length scale and rather in two dimensions. One-monolayer-deep vacancy islands evolve at potentials where C_2_H_4_ starts forming (−0.61 V_RHE_; see Figure 4d–f). Structural and morphological changes lead to pronounced restructuring that appears as material removal and readdition in the same surface layer; i.e., no protrusions and visibly less undercoordinated sites are forming. Interestingly, smaller (∼3.5 ± 0.4 nm in size) Cu ad-islands are observed upon stepping the potential back to the reference value of −0.18 V_RHE_ (see Supporting Figure S9), which are vanishing after an extended time period.

The substantially different types of restructuring of Cu(100) and Cu(111) after potential steps to the CORR onsets raise the puzzle of whether those structural rearrangements influence the reaction mechanism. The dominant question in this respect is where these differences in the reconstruction behavior arise from. To find answers to this question, experiments without CO in the electrolyte have been conducted (Supporting Figures S10 and S11). These experiments show that, on Cu(100) (Figure S10), dark patches form directly after the potential step to −0.48 V_RHE_. The occurrence of these patches is reversible; they disappear upon a potential increase back to −0.18 V_RHE_. Without CO in the electrolyte, no heavy reconstruction, i.e., protrusion formation, was found on Cu(100) at cathodic potentials. This strongly indicates that the presence of CORR intermediates is required to form and stabilize the small ad-islands that were observed under the CORR conditions (see Figure 4a–c).

The same reference EC-STM experiments on Cu(111) without CO in 0.1 M NaOH upon a potential step to −0.61 V_RHE_ (Supporting Figure S11) show a very similar type of restructuring as imaged with CO in solution. A rearrangement of surface atoms is visible where larger ad- and vacancy islands are formed. This leads to the assumption that the interaction of the reaction products with the Cu(111) surface is less influential, while the CO-free electrolyte and the HER alone have the potential to effectively restructure it.

A possible explanation for this behavior goes back to the interaction and reactivity of the interfacial water with the two different surfaces. On Cu(100), no reconstruction is observed, but only the occurrence of dark areas is observed. This type of dark area could be attributed to the presence of OH^–^, as has previously been observed upon OH adsorption on the Cu surface at potentials positive of the pzfc.^45^ In the system examined here, OH^–^ can be formed due to the dissociation and the subsequent reduction of H_2_O_dl_ as also indicated by the EC-IRRAS results. On Cu(111), the interfacial water reduction can apparently cause a surface reconstruction, even if no CO or CORR intermediates are present at the interface. This corroborates the strong interaction and high reactivity of interfacial water, which is also observed for Cu(111) with DEMS and EC-IRRAS. It further indicates that it is this interaction that largely determines the restructuring of the (111) surface and not the presence of CORR intermediates or products as in the case of Cu(100), thus underscoring the relevance of the interfacial water reactivity on the CORR for different Cu surfaces.

## Conclusion

In summary, we have discussed the importance of the reactivity of interfacial water at Cu single-crystal surfaces regarding the CORR. The relative activities toward CH_4_ and C_2_H_4_ formation can be increased by lowering the H* adsorption barrier, as provided by (defect-rich) Cu(111) surfaces. This seems to be even more important than CO adsorption, which has not been directly observed on either surface at the investigated potentials. The formation of *CHO, one key intermediate in the CORR pathway, is enhanced on Cu(111) due to a higher H* coverage. This results in the highest CORR activities on this surface. As the hydrogenation of *CHO on Cu(111) appears fast, more CH_4_ than on Cu(100) is formed. The small H* coverage on Cu(100) inhibits the fast hydrogenation of *CHO to CH_4_ and leads to the selective reaction of *CHO to C_2_H_4_.

The HER and the CORR induce a surface reconstruction at Cu(100), where small protruding Cu ad-islands are immediately formed and stabilized, as observed *in situ* with EC-STM. No reconstruction is found without CO in the solution. On Cu(111), a rearrangement of surface atoms on a larger length scale is observed in the CO-containing and CO-free electrolyte, where no protrusions form. It is very likely that in the case of Cu(111), the interaction of the surface with interfacial water is the main driving force for the restructuring under the CORR conditions. The interaction of any CORR product or intermediate with the surface seems to be less dominant. These findings highlight the essential role of the interfacial water reactivity in the CORR in alkaline media on Cu.

## Materials and Methods

### Electrochemistry and Electrode Materials

A 0.1 M NaOH solution prepared from NaOH monohydrate (99.99%, Merck, Germany) and ultrapure water (Milli-Q, Merck, Germany) saturated with CO (99.995%, Linde, Germany) served as the electrolyte (pH = 13). To avoid possible dissolution of glassware, the electrolyte was prepared and stored in a polytetrafluoroethylene (PTFE) beaker, which was cleaned by immersion in an aqueous KMnO_4_ (98%, Alfa Aesar, U.S.) solution for 20 min, subsequently washed with diluted piranha solution (H_2_SO_4_ (96%, Merck, Germany)/H_2_O_2_ (Perhydrol, 30%, Merck Germany), 3:1), and thoroughly rinsed with Milli-Q water before use.

The reference and counter electrodes were PTFE-bound activated carbon electrodes supported on carbon rods, which served as current collectors.^46^ For the activated carbon electrodes, 1.4 g of Carbon Black (99%, Cabot, U.S.) and 2.0 g of PTFE suspension (60% in water, Merck, Germany) were mixed with 40 mL of 1:3 solution 2-propanol (96%, VWR Chemicals, U.S.)/H_2_O. The solvent was evaporated under vigorous stirring to obtain a plastic dough.

All potentials in this paper are given versus the reversible hydrogen electrode (RHE).

The Cu single crystal electrodes were mechanically polished using diamond suspensions (3, 1, and 0.25 μm diameter, ESCIL, France), electropolished in 60% H_3_PO_4_ (from 80% H_3_PO_4_, Merck, Germany) at 1.8 V vs a Cu counter electrode, subsequently washed with 10% H_3_PO_4_ and ultrapure water, and dried in an argon (Ar, 99.990%, Messer, Austria) stream. Afterward, the single crystals were thermally annealed under a constant hydrogen (H_2_; 99.990%, Messer, Austria) flow at 840 °C overnight. These samples are denoted as “quasi-ideal” in this work. To increase the surface defect concentration, the annealed single crystals were electrochemically polished in 60% H_3_PO_4_ for 30 s at 1.8 V vs a Cu counter electrode and thoroughly rinsed with 10% H_3_PO_4_ and ultrapure water. These samples are denoted as “defect-rich” in this work.

### Electrochemical Scanning Tunneling Microscopy (EC-STM)

All EC-STM experiments were performed with a Keysight 5500 scanning probe microscope inside an Ar-filled glovebox (MB 200 MOD, MBraun, Germany). The Cu single crystals were mounted in a custom-made EC-STM cell with PTFE-bound activated carbon quasi-reference and counter electrodes.^46^ The Cu single crystals were immersed at the OCP and then reduced to remove the native oxide layer. The electrolyte was deaerated with Ar prior to each experiment. CO (99.995%, Linde, Germany) saturation of the electrolyte was performed inside the glovebox via an all-copper gas dosing system to prevent iron and nickel contamination. For the processing and representation of all EC-STM images, WSxM was used.^47^

### Differential Electrochemical Mass Spectrometry (DEMS)

Differential electrochemical mass spectrometry was carried out with an HPR-40 system (Hiden Analytical, U.K.), equipped with a quadrupole mass filter and a secondary electron multiplier detector (SEM). Electron ionization was carried out with 70 eV of energy, and all generated ions were accelerated with a cage voltage of 3 V except for hydrogen ions, where the cage potential was set to 2 V to avoid detector saturation. The multiplier potential of SEM was 932 V. A PTFE membrane with a 200 nm pore size and 35 μm thickness (Cobetter, China) supported on a stainless-steel frit served as a separator between the electrochemical cell and the high vacuum compartment of the mass spectrometer. For all experiments, a PEEK flow cell (Hiden Analytical, U.K.) with a constant electrolyte flow rate of 50 μL min^–1^ was used. Further information on the setup and the delay time can be found elsewhere.^48−50^ A Metrohm Autolab (PGSTAT 302N) was used as potentiostat. The scan rate was 2 mV s^–1^, and the *m*/*z* signals of 2 (hydrogen, H_2_^+^), 15 (methane, CH_3_^+^) and 26 (ethylene, C_2_H_2_^+^) were simultaneously recorded.

### Electrochemical Infrared Spectroscopy (EC-IRRAS)

EC-IRRAS experiments were performed on a VERTEX 70v spectrometer (Bruker, U.S.) in a home-built three-electrode spectro-electrochemical cell made of polychlorotrifluoroethylene mounted into an external chamber (XSA, Bruker, U.S.) equipped with a linear polarizer (Edmund Optics, Germany). Thereby, a liquid-nitrogen-cooled mercury cadmium telluride (MCT) photodetector was used. Internal reflection was achieved by employing two adjustable gold mirrors reflecting the incoming IR beam onto the working electrode pressed onto a CaF_2_ hemisphere. Both reference and counter electrodes were activated carbon prepared as described above,^46^ supported on flame-annealed carbon rods, respectively.

## Data Availability

The raw data supporting the conclusions of this article will be made available by the corresponding author upon request, without undue reservation.

## Supporting Information Available

DEMS results for the CO reduction and HER on quasi-ideal and defect-rich Cu(111) and Cu(100); zooms into the *m*/*z* signals of C_2_H_4_ for quasi-ideal and defect-rich Cu(100) and Cu(111); onset potential determination for the product formation in CO-saturated and Ar-purged electrolyte; RFS values for the formation of H_2_, CH_4_, and C_2_H_4_; Tafel slopes of the *m*/*z* = 2 signal for quasi-ideal and defect-rich Cu(100) and Cu(111) in an Ar-purged electrolyte; additional EC-STM measurements for the reversibility of Cu(100) and Cu(111) during the CO reduction; cluster size distribution of Cu islands at CO reduction potentials; EC-STM measurements of Cu(100) and Cu(111) during the HER (PDF)
