# Supplementary material for: The Relevance of the Interfacial Water Reactivity for Electrochemical CO Reduction on Copper Single Crystals
Source: ACS Catal. 2024 Jan 8;14(2):1098–106. doi: 10.1021/acscatal.3c02700 (PMC10806897; doi:10.1021/acscatal.3c02700)
Supplement: Supplementary file 1 — cs3c02700_si_001.pdf [file cs3c02700_si_001.pdf]

Supporting Information

The relevance of the interfacial water reactivity for electrochemical CO reduction on copper single crystals

Daniel Winkler[1†], Matthias Leitner[1†], Andrea Auer[1] and Julia Kunze-Liebhäuser[1*]

[1]Department of Physical Chemistry, University of Innsbruck, Innrain 52c, 6020 Innsbruck, Austria

[†]These authors have contributed equally to this work and share first authorship

*** Correspondence:**
Julia Kunze-Liebhäuser
Julia.Kunze@uibk.ac.at

1 Onset of the CO electroreduction

The onset potentials of the formation of all CO reduction products, i.e. hydrogen (H_2), methane (CH_4) and ethylene (C_2H_4), were determined from the mass spectrometric linear sweep voltammograms (LSVs) (Figures S1+S2). The ionic currents in the potential region between -0.05 and -0.30 V_{RHE}, where no reaction is expected to occur, were used to calculate the signal background values and their standard deviations. The onset of the reaction is defined as the potential at which the ionic current values of the different products exceed three times the value of the standard deviation of the background value. The onset potentials are summarized in Supporting Tables S1 (CO reduction). For the hydrogen evolution reaction (HER) in Ar-saturated 0.1 M NaOH the onset potentials were determined from the mass spectrometric LSVs shown in Figure S5 and are listed in Supporting Table S2.

Supporting Table S1. Onset potentials of the H_2, CH_4 and C_2H_4 formation at quasi-ideal Cu(100) and Cu(111) and defect-rich Cu(100) and Cu(111).

	E_{onset} H_2 / V_{RHE}	E_{onset} CH_4 / V_{RHE}	E_{onset} C_2H_4 / V_{RHE}
**quasi-ideal Cu(100)**	-0.63	-0.70	-0.56
**defect-rich Cu(100)**	-0.44	-0.86	-0.43
**quasi-ideal Cu(111)**	-0.65	-0.61	-0.74
**defect-rich Cu(111)**	-0.70	-0.65	-0.54

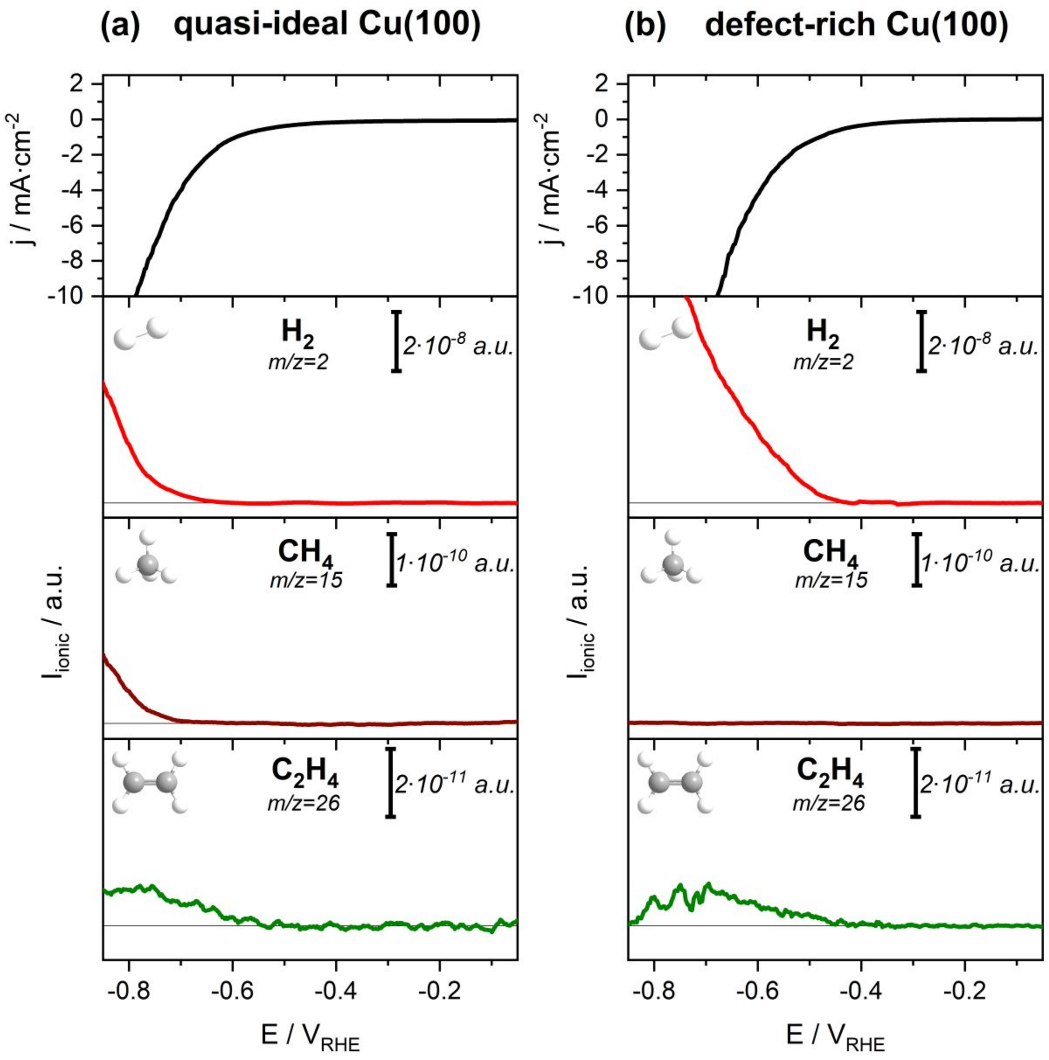

Supporting Figure S1. LSVs (top panels) and mass spectrometric responses for H_2^+ (m/z=2), CH_3^+ (m/z=15) and $C_2H_2^+$ (m/z=26) during the CO electroreduction in 0.1 M NaOH on (a) quasi-ideal Cu(100) and (b) defect-rich Cu(100). Scan Rate: 2 mV/s.

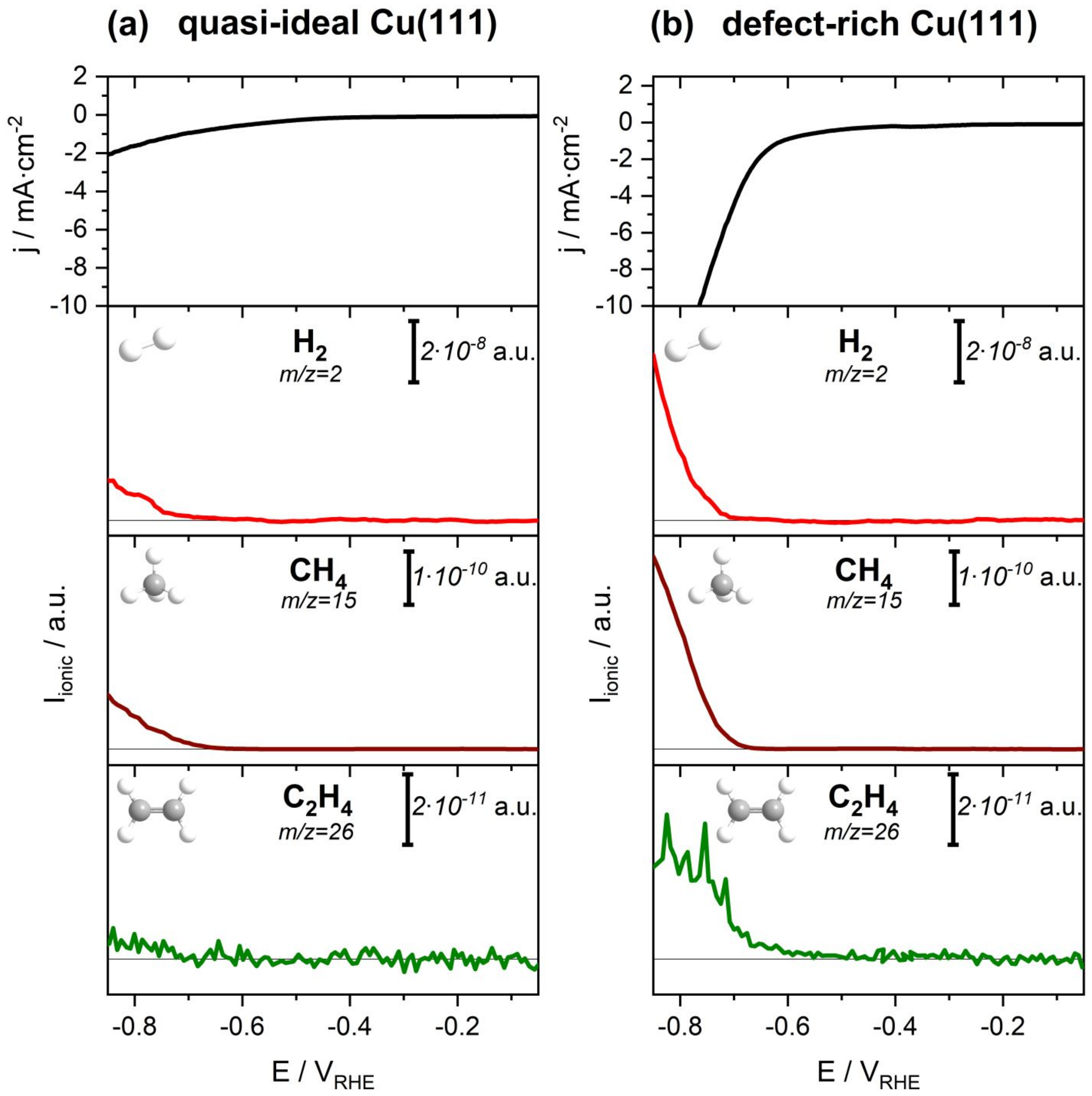

Supporting Figure S2. LSVs (top panels) and mass spectrometric responses for H_2^+ (m/z=2), CH_3^+ (m/z=15) and $C_2H_2^+$ (m/z=26) during the CO electroreduction in 0.1 M NaOH at (a) quasi-ideal Cu(111) and (b) defect-rich Cu(111) . Scan Rate: 2 mV/s.

2 Relative Faraday selectivities

The relative Faraday selectivities for the formation of C_2H_4 were calculated from the mass spectrometric responses of all products (H_2, CH_4 and C_2H_4) in analogy to Reference 1.

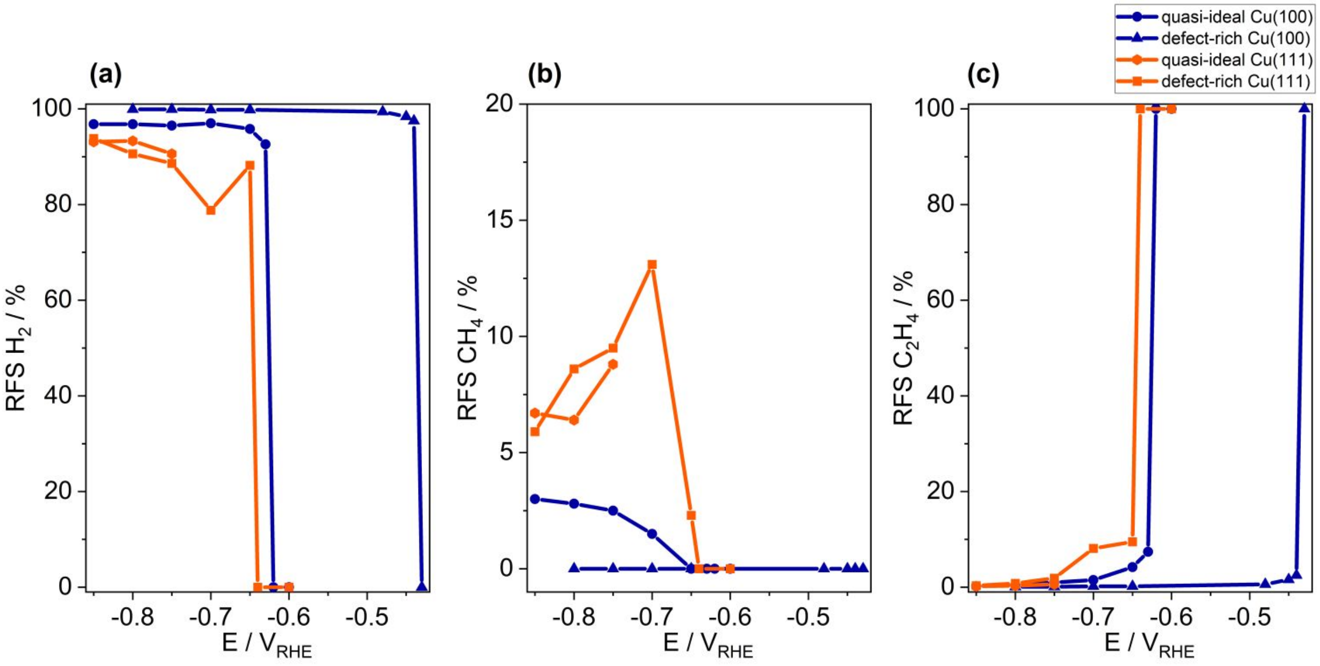

Supporting Figure S3. Relative Faraday selectivity values for the formation of H_2 (a), CH_4 (b) and C_2H_4 (c) considering the ionic currents in the mass spectrometric LSVs of all recorded products.

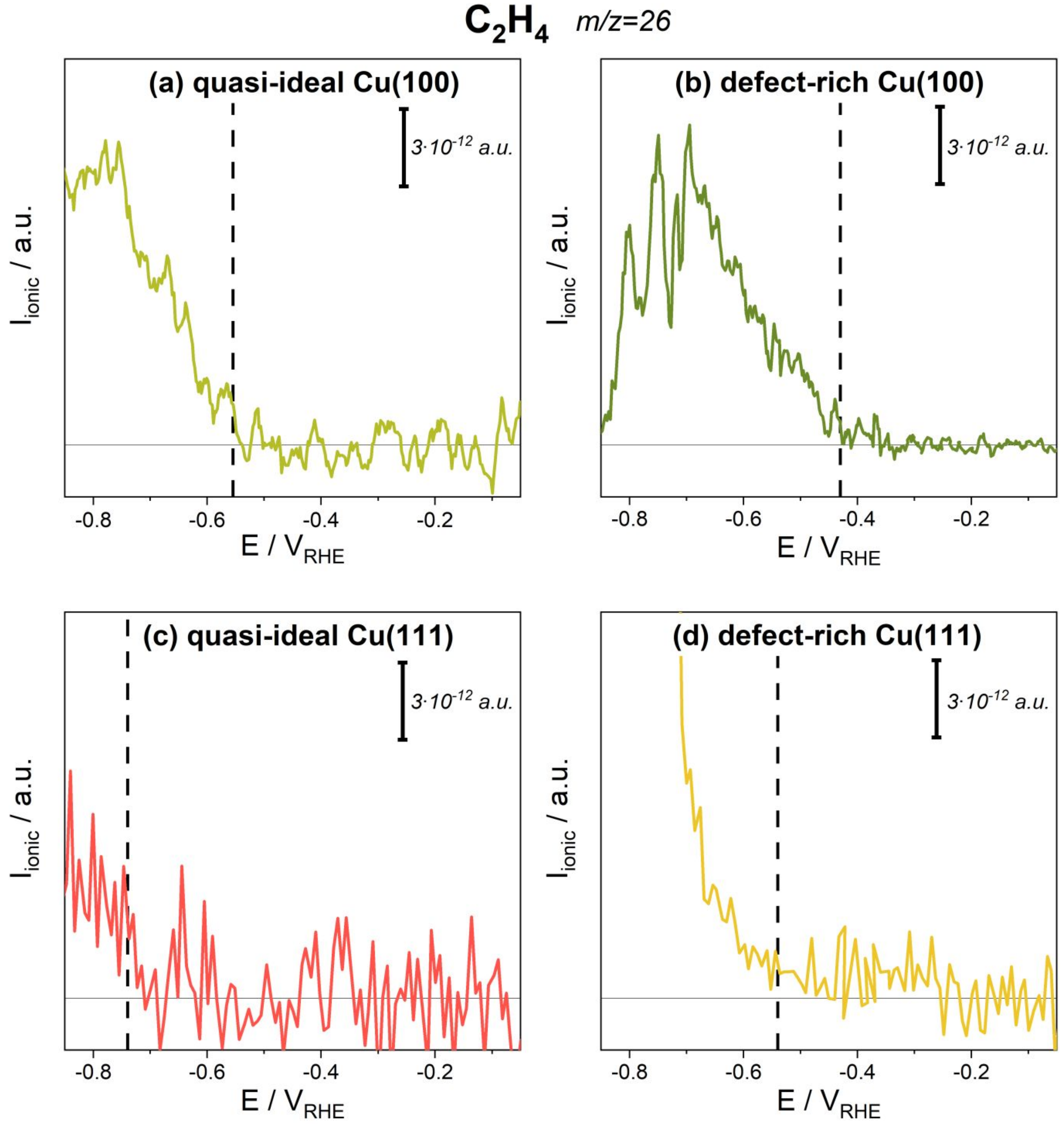

Supporting Figure S4. Zoom into the mass spectrometric responses of the LSVs for the ethylene signals of quasi-ideal and defect-rich Cu(100) and quasi-ideal and defect-rich Cu(111) during the CO electroreduction in 0.1 M NaOH. The onset potentials for the ethylene formation (see Supporting Table S1) are marked with dashed lines in the respective panel. Scan Rate 2 mV/s.

3 HER onset potentials and kinetics in CO-free electrolyte

The HER onset potential in CO-free 0.1 M NaOH electrolyte as determined from the mass spectrometric LSVs are shown in Figure S5. No significant difference between the onsets of defect-rich and quasi-ideal surfaces are observed, only the Faraday currents and the mass spectrometric responses are higher for the defect-rich surfaces, which can be correlated to the increased surface areas compared to the quasi-ideal surfaces. This evidences that the introduction of surface defects does not influence the HER onset potentials. The CO electroreduction and its product distribution, however, strongly depend on structural motifs at the surface, which are altered by the induction of defects. The Tafel-Plot of the ionic currents of m/z=2 (H_2^+) are shown in Figure S6. All samples show a Tafel-slope of 120-130 mV/dec indicating very slow HER kinetics, with the Volmer step being the rate determining step.

Supporting Table S2. HER onset potentials at Cu(100) and Cu(111) in CO-free 0.1 M NaOH (pH=13).

	**E_{onset} H_2 / V_{RHE}**
**quasi-ideal Cu(100)**	-0.44
**defect-rich Cu(100)**	-0.42
**quasi-ideal Cu(111)**	-0.57
**defect-rich Cu(111)**	-0.54

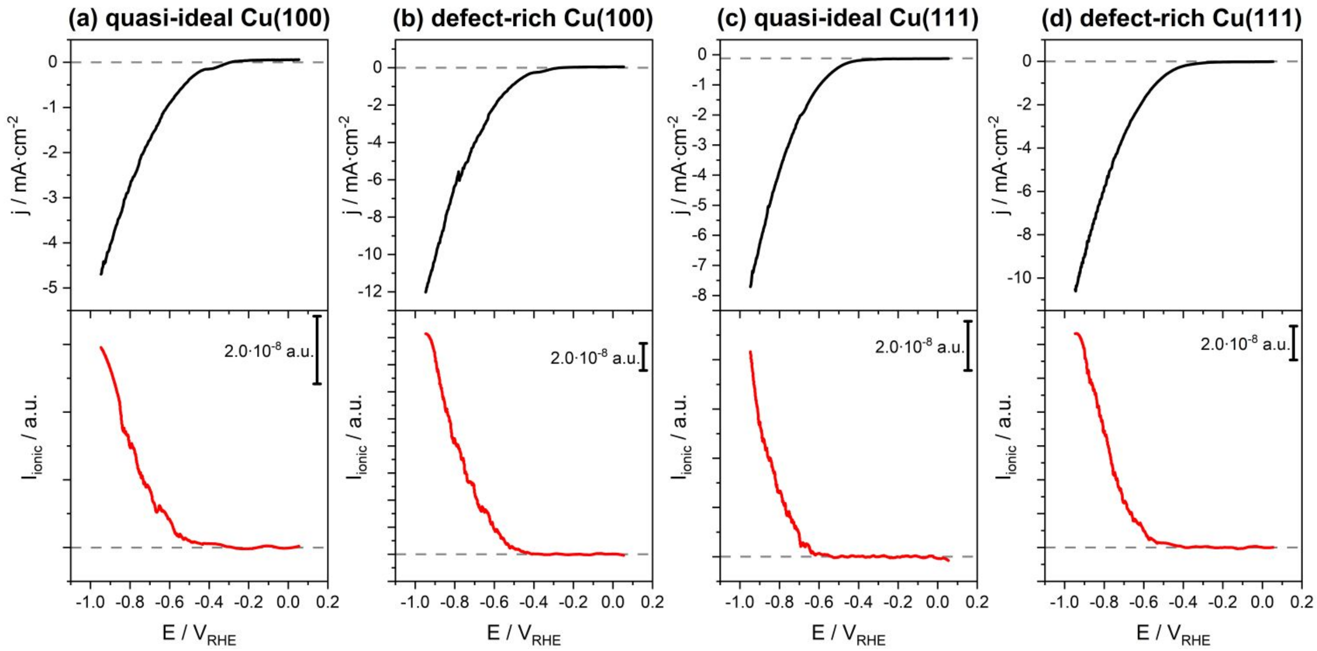

Supporting Figure S5. LSVs (top panels) and mass spectrometric responses for H_2^+ (m/z=2 during the HER in CO-free, Ar-purged 0.1 M NaOH on (a) quasi-ideal Cu(100), (b) defect-rich Cu(100), (c) quasi-ideal Cu(111) and (d) defect-rich Cu(111), Scan Rate: 2 mV/s.

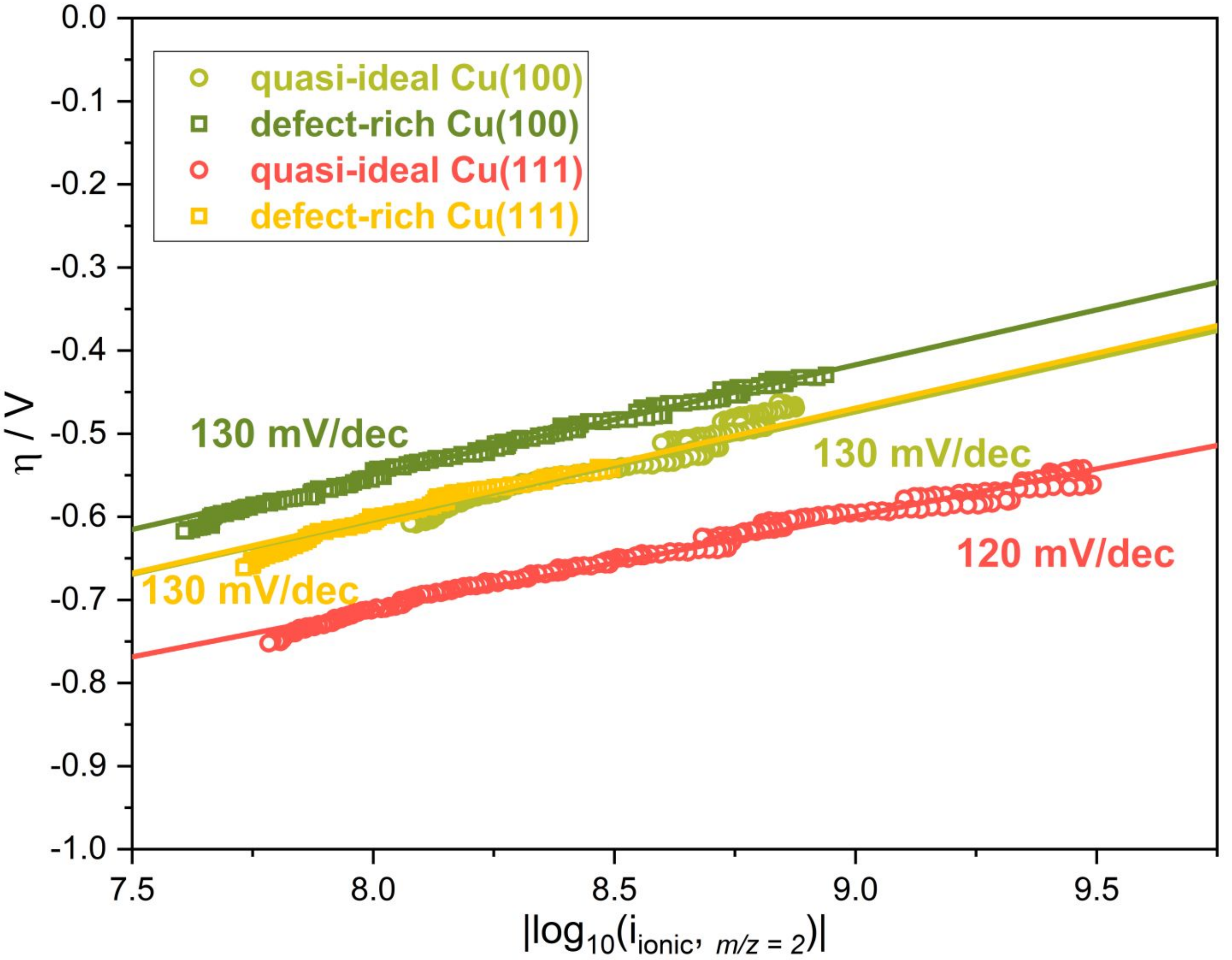

Supporting Figure S6. Tafel plots for the HER derived from the ionic currents of hydrogen (m/z=2) measured with DEMS for quasi-ideal and defect-rich Cu(100) and Cu(111) in Ar-purged 0.1 M NaOH. Scan rate 2 mV/s.

4 Electrochemical Scanning Tunneling Microscopy (EC-STM) of Cu(hkl) under CORR conditions

The size analysis of the Cu ad-islands on Cu(100), generated with the grain analysis tool and the watershed algorithm in Gwyddion, [2] is shown in Supporting Figure S7. The most common clusters exhibit diameters of 0.52, 1.35 and 2.19 nm, with areas of 0.65, 1.09 and 1.52 nm^2 projected by the algorithm.

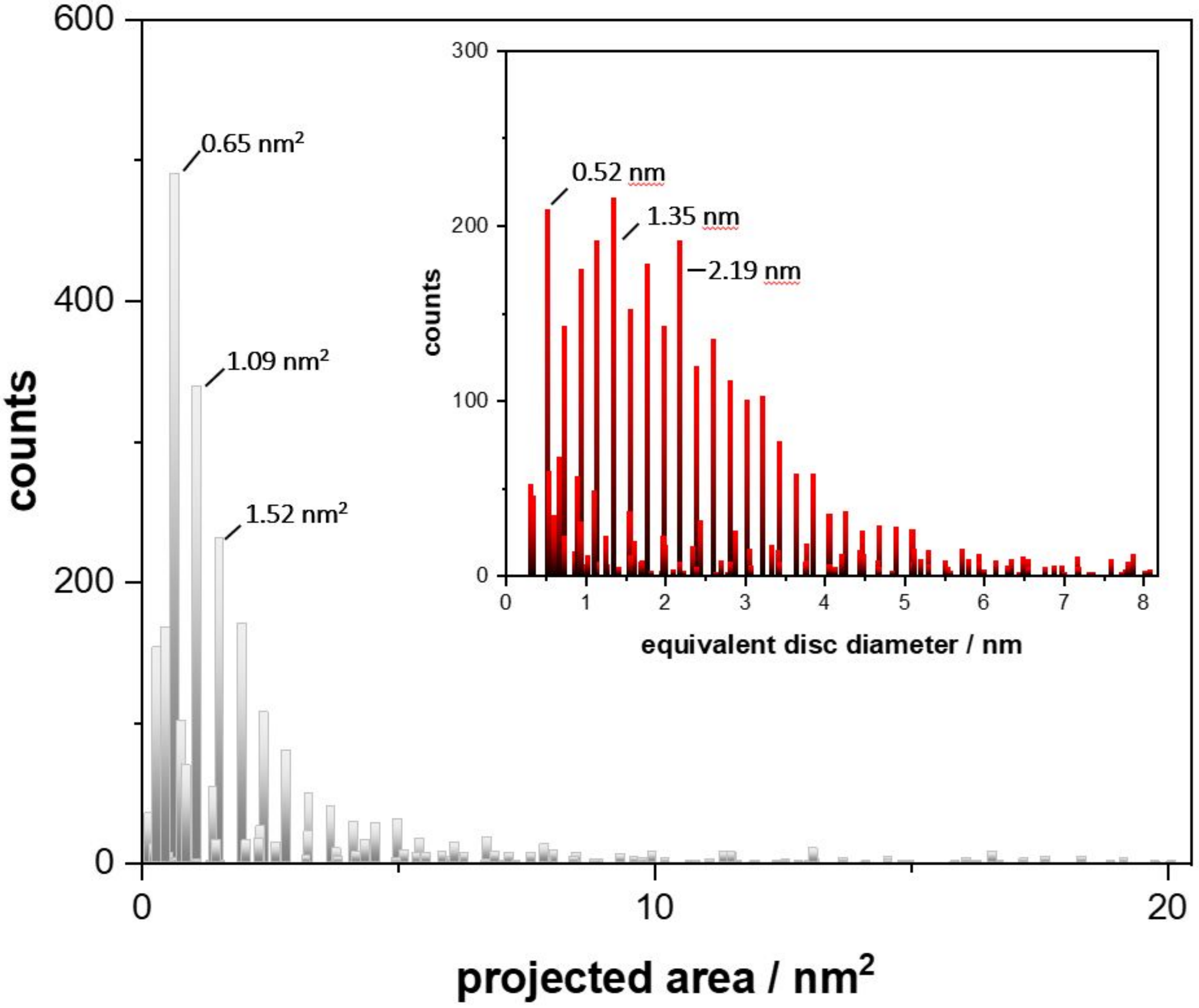

Supporting Figure S7. Cluster size distribution of ad-islands on Cu(100) with a projected size (areas of elevation marked by the watershed algorithm) at the CO reduction potential of -0.48 V_{RHE}. The inset shows the equivalent disc diameter.

The reversibility of the reconstruction observed at CO reduction potentials of -0.48 V_{RHE} for Cu(100) (Supporting Figure S8) and -0.61 V_{RHE} for Cu(111) (Supporting Figure S9) in CO saturated electrolyte and -0.48 V_{RHE} for Cu(100) (Supporting Figure S10) and -0.61 V_{RHE} for Cu(111) (Supporting Figure S11) in Ar saturated electrolyte is monitored over one hour after jumping back to the reference potential of -0.18 V_{RHE}.

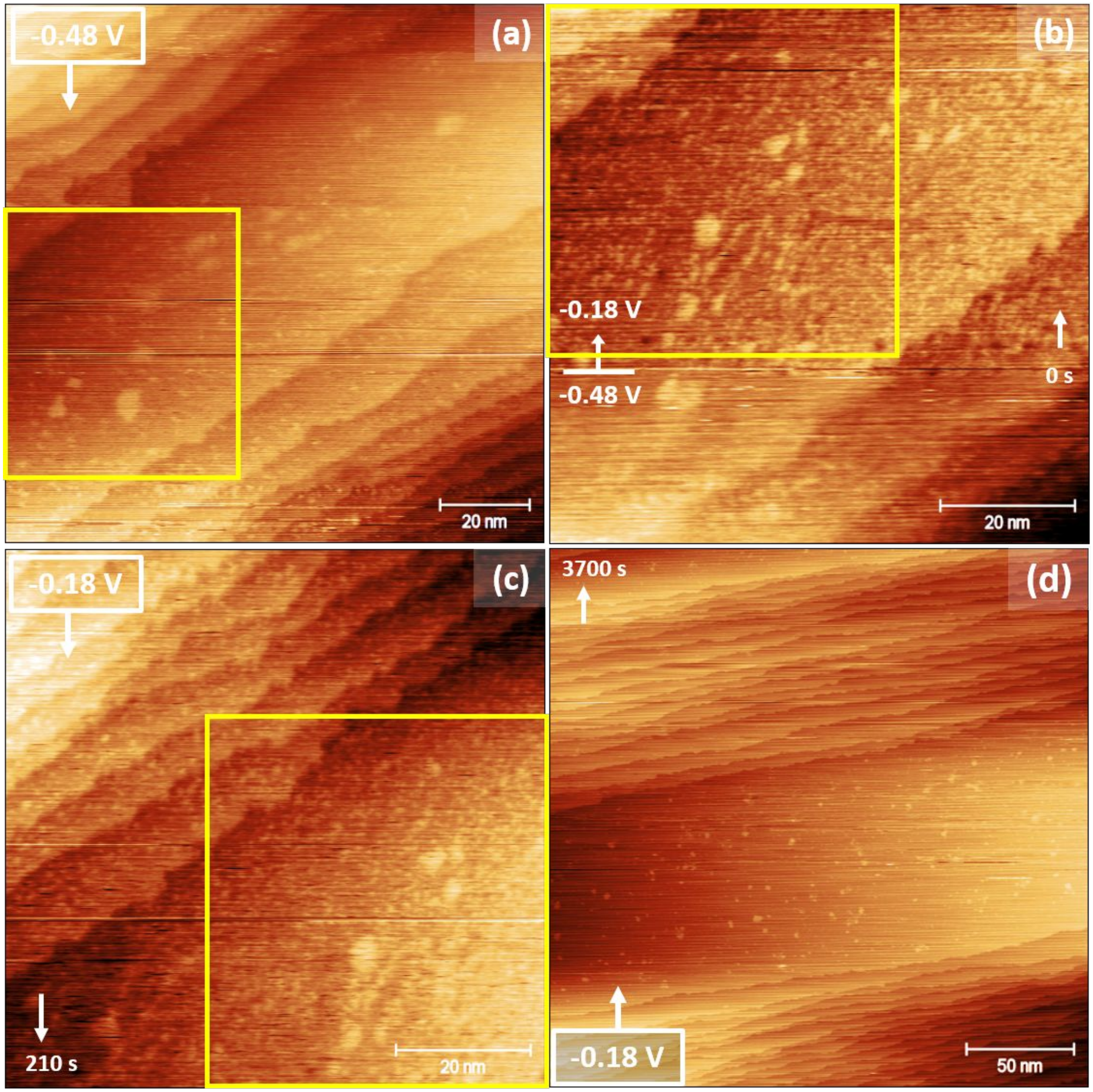

Supporting Figure S8. Cu(100) after a potential step from -0.48 V_{RHE} (a) back to the reference potential of -0.18 V_{RHE} as indicated in (b), illustrating the reversibility of the restructuring process when applying the reference potential for 210 s (c) and 3700 s (d). Image sizes: 120×120 nm^2 (a), 80×80 nm^2 (b, c), 250×250 nm^2 (d); I_{tun} = 1.8 nA (a, d) and 4 nA (b, c); E_{tip} = 0.27 V_{RHE}. Yellow squares indicate the same area sections.

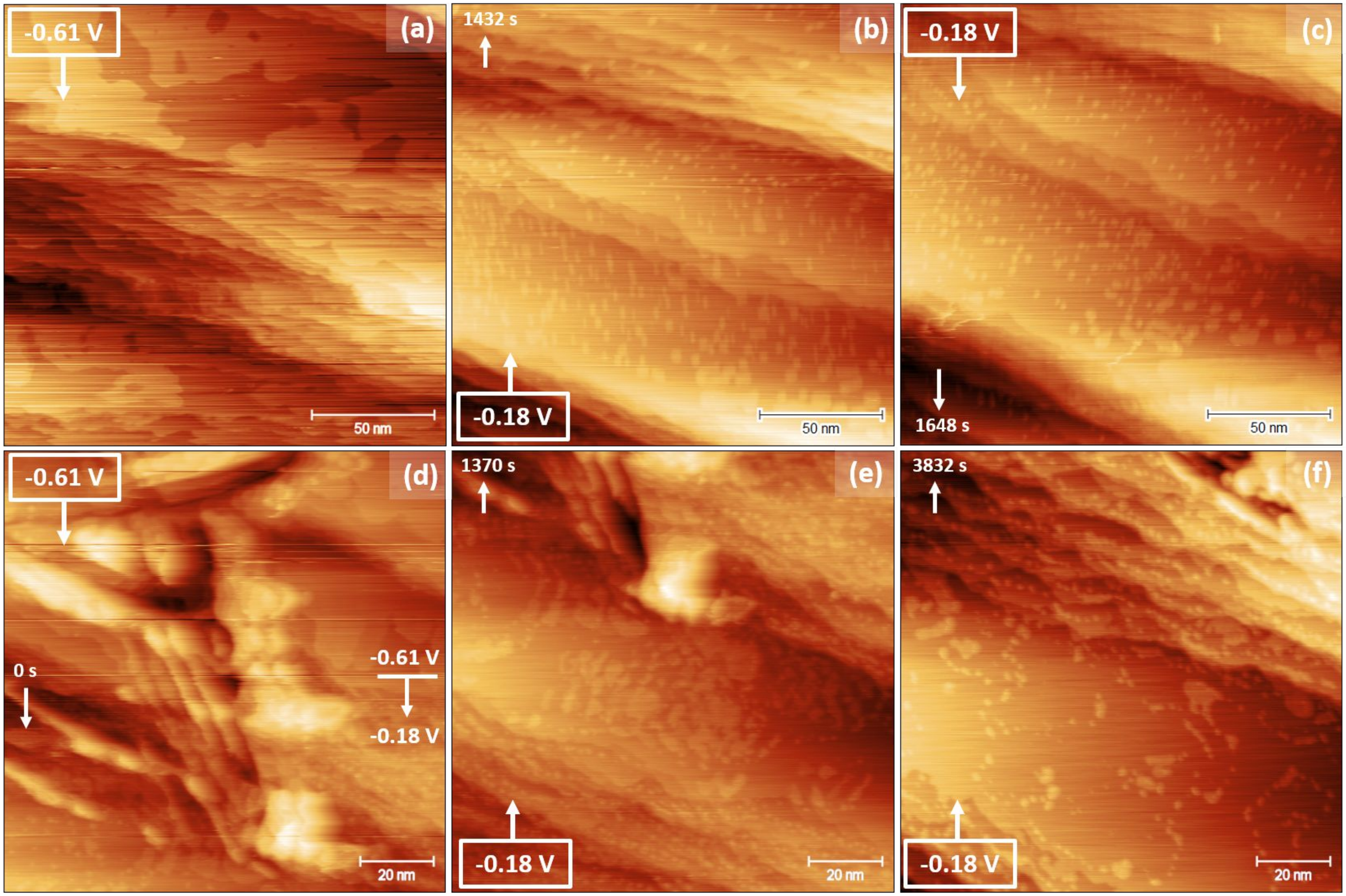

Supporting Figure S9. Cu(111) in CO saturated NaOH (pH=13) after 613 s at -0.61 V_{RHE} (panel a), and after (b) 1432 s and (c) 1648 s after the potential step back to the reference potential of -0.18 V_{RHE}. Panels d-f show the surface of a different sample displayed over a longer timescale, where the potential step back to the reference potential is indicated in image (d), the surface at 1370 s (e) and 3832 s (f) after reduction show a diminished number of ad-islands. Image sizes: 180×180 nm^2 (a-c), 120×120 nm^2 (d-f); I_{tun} = 3.5 nA (a-c), 5 nA (d-f); E_{tip} = 0.32 V_{RHE} (a-c), 0.22 V_{RHE} (d-f).

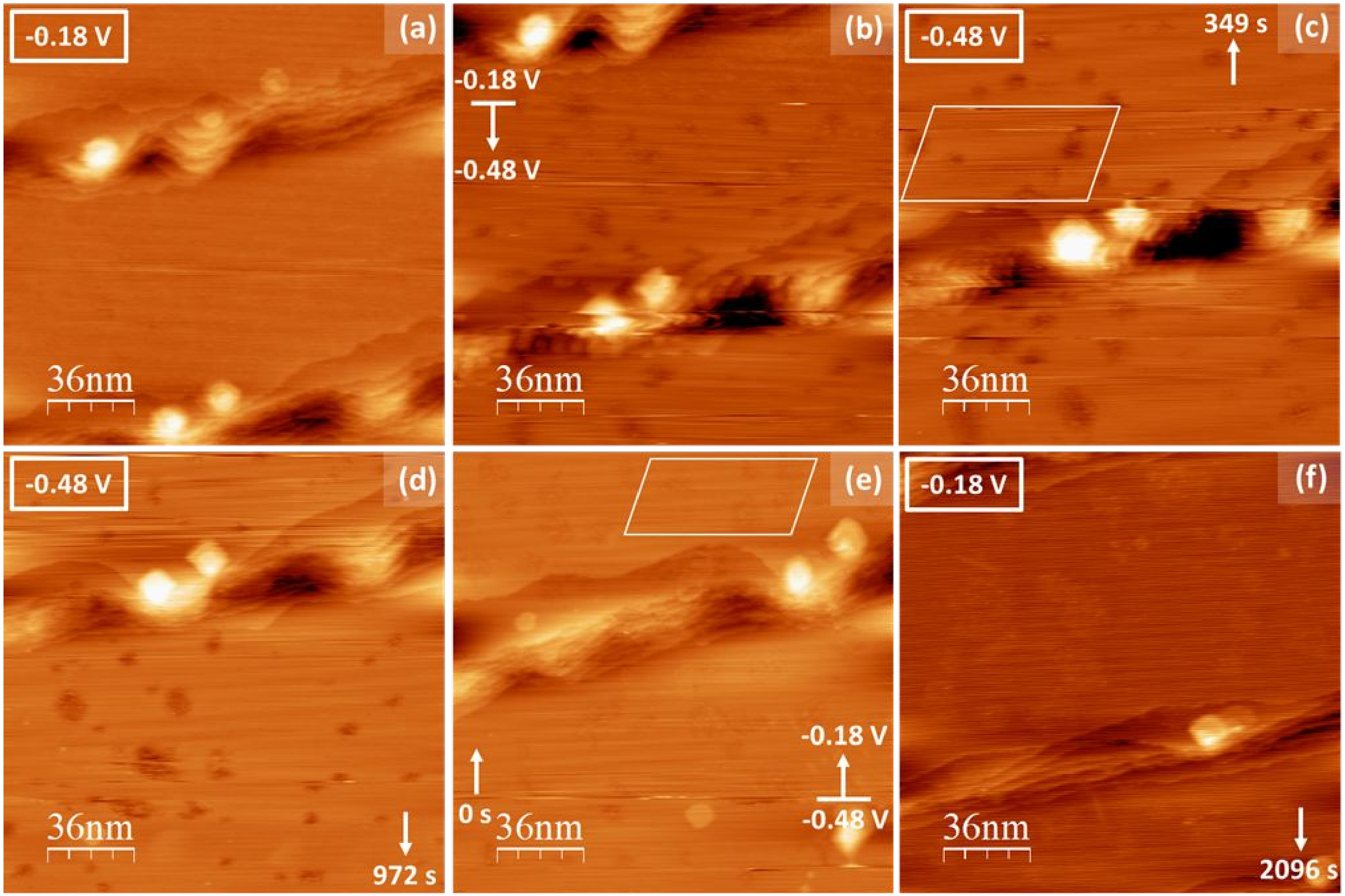

Supporting Figure S10. *In situ* EC-STM images of Cu(100) during HER conditions in Ar saturated 0.1 M NaOH (pH=13). The bare Cu(100) surface (a) changes after a potential step to hydrogen evolution potentials (i.e. -0.48 V_{RHE}) (b-d); dark areas indicate OH adsorption. [3] Note that the region marked with white rectangles in panels (c) and (e) display the same spot on the surface. The terraces are 'healing' after stepping the potential to -0.18 V_{RHE}. Image sizes: 180×180 nm^2, I_{tun} = 1.5 nA, E_{tip} = 0.23 V_{RHE}.

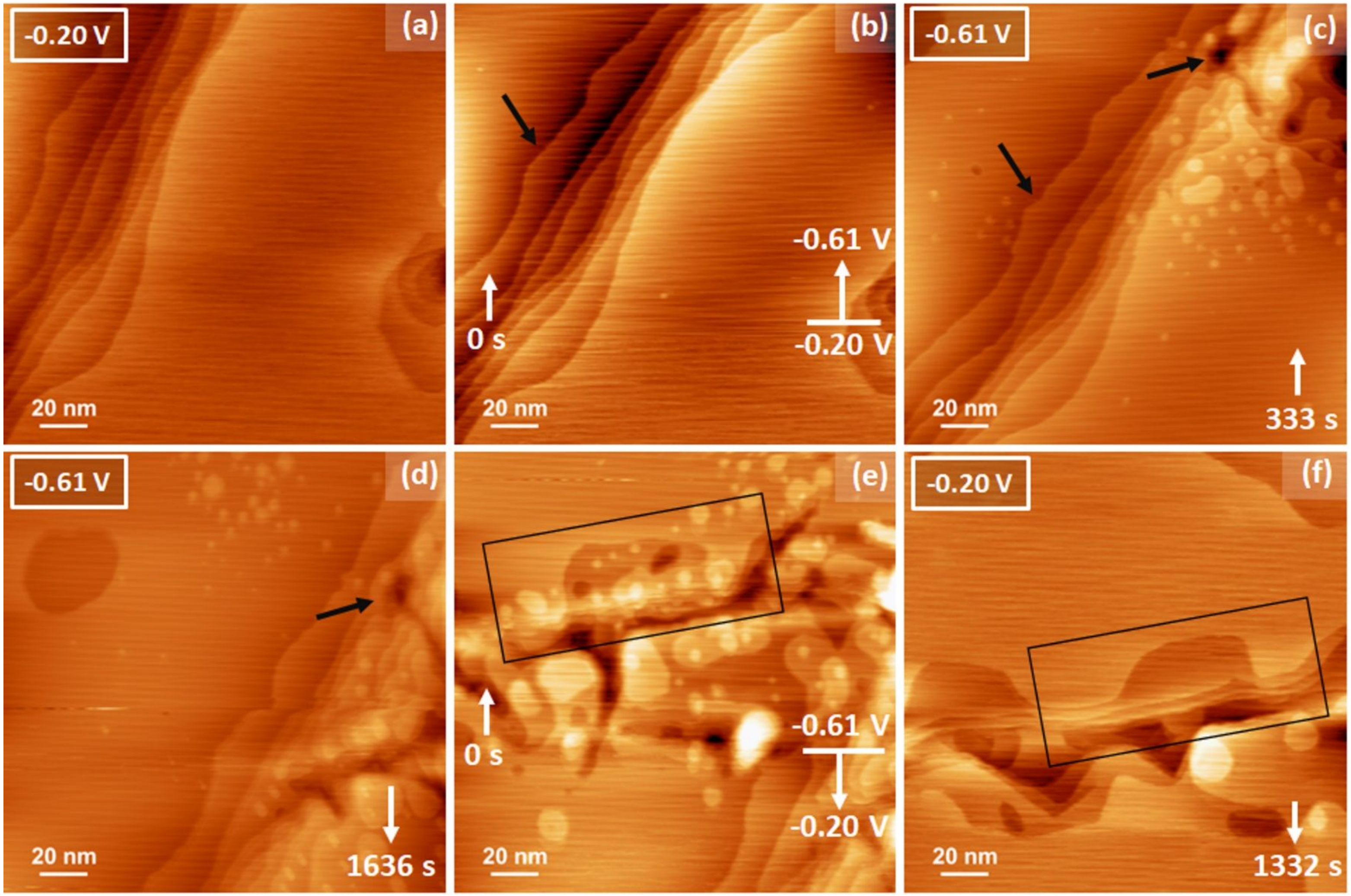

Supporting Figure S11. *In situ* EC-STM images of Cu(111) before (a) and after (b) a potential step to -0.61 V_{RHE} in Ar saturated 0.1 M NaOH (pH=13). The unreconstructed Cu(111) surface at the reference potential of -0.20 V_{RHE} (a), exhibits structural changes after the potential step to hydrogen evolution potentials, i.e. -0.61 V_{RHE} for 333 s (c) and 1636 s (d). The same positions are marked with black arrows. After 2066 s, a potential step back to -0.20 V_{RHE} was executed (panel e). Panel f shows the surface when the potential is held for another 1332 s at -0.20 V_{RHE}. Black rectangles show the same areas in (e) and (f). White arrows indicate the slow scan direction. Image sizes: 180×180 nm^2, I_{tun} = 2.5 nA, E_{tip} = 0.17 V_{RHE}.

5 References

(1) Reske, R.; Duca, M.; Oezaslan, M.; Schouten, K. J. P.; Koper, M. T. M.; Strasser, P. Controlling Catalytic Selectivities during CO_2 Electroreduction on Thin Cu Metal Overlayers. *J. Phys. Chem. Lett.* **2013**, *4* (15), 2410–2413. DOI: 10.1021/jz401087q.

(2) Nečas, D.; Klapetek, P. Gwyddion: an open-source software for SPM data analysis. *Open Phys.* **2012**, *10* (1). DOI: 10.2478/s11534-011-0096-2.

(3) Kunze-Liebhäuser, J. Electrochemical Scanning Tunneling Microscopy Studies of Copper Oxide Formation—A Review. In *Encyclopedia of Interfacial Chemistry*; Elsevier, 2018; pp 107–120. DOI: 10.1016/B978-0-12-409547-2.14135-6.
